# Morphological switch to a resistant subpopulation in response to viral infection in the bloom-forming coccolithophore *Emiliania huxleyi*

**DOI:** 10.1371/journal.ppat.1006775

**Published:** 2017-12-15

**Authors:** Miguel José Frada, Shilo Rosenwasser, Shifra Ben-Dor, Adva Shemi, Helena Sabanay, Assaf Vardi

**Affiliations:** 1 Department of Plant and Environmental Sciences, Weizmann Institute of Science, Rehovot, Israel; 2 Bioinformatics and Biological Computing Unit–Department of Biological Services, Weizmann Institute of Science, Rehovot, Israel; 3 Department of Chemical Research Support, Weizmann Institute of Science, Rehovot, Israel; Georgia Tech, UNITED STATES

## Abstract

Recognizing the life cycle of an organism is key to understanding its biology and ecological impact. *Emiliania huxleyi* is a cosmopolitan marine microalga, which displays a poorly understood biphasic sexual life cycle comprised of a calcified diploid phase and a morphologically distinct biflagellate haploid phase. Diploid cells (2N) form large-scale blooms in the oceans, which are routinely terminated by specific lytic viruses (EhV). In contrast, haploid cells (1N) are resistant to EhV. Further evidence indicates that 1N cells may be produced during viral infection. A shift in morphology, driven by meiosis, could therefore constitute a mechanism for *E*. *huxleyi* cells to escape from EhV during blooms. This process has been metaphorically coined the ‘Cheshire Cat’ (CC) strategy. We tested this model in two *E*. *huxleyi* strains using a detailed assessment of morphological and ploidy-level variations as well as expression of gene markers for meiosis and the flagellate phenotype. We showed that following the CC model, production of resistant cells was triggered during infection. This led to the rise of a new subpopulation of cells in the two strains that morphologically resembled haploid cells and were resistant to EhV. However, ploidy-level analyses indicated that the new resistant cells were diploid or aneuploid. Thus, the CC strategy in *E*. *huxleyi* appears to be a life-phase switch mechanism involving morphological remodeling that is decoupled from meiosis. Our results highlight the adaptive significance of morphological plasticity mediating complex host–virus interactions in marine phytoplankton.

## Author summary

This study assesses the interplay between the globally distributed microalga *Emiliania huxleyi* and its specific lytic viruses, EhV, which drive the termination of vast oceanic blooms. *E*. *huxleyi* is characterized by a biphasic life cycle that alternates between morphologically dissimilar diploid and haploid cells. Here, we show that during viral infection, the bloom-forming diploid cells that are sensitive to EhV can produce virus-resistant cells. These latter cells are morphologically similar to the haploid phase but have diploid or aneuploid genomes. Therefore, a mechanism that mediates morphological remodeling appears to be activated during viral infection, enabling *E*. *huxleyi* to escape EhV. These results provide novel insights into morphological plasticity and viral resistance in marine phytoplankton, while highlighting the complexity of host–virus interactions in the oceanic microbial realm.

## Introduction

The life cycle of an organism represents a multitude of cellular stages connected by reproductive processes. These complex chains of events have been selected over a long evolutionary history and represent a key feature underlying species ecology [1,2]. Thus, unraveling all cellular stages and the factors driving life-phase transitions will enhance our understanding of species' functional roles and their adaptive responses to environmental variations. With the exception of a few model organisms and human parasites, however, little is known about the life cycle of microbial eukaryotes, which paradoxically represent the vast majority of extant eukaryotic diversity [3]. This knowledge gap is exemplified by marine phytoplankton, which comprise a highly diverse assemblage of phototrophic species that make an important contribution to the base of the marine food web and global biogeochemical processes [2,4].

The coccolithophore *Emiliania huxleyi* (Lohmann) Hay and Mohler (Prymnesiophyceae) is a globally distributed marine microalga that forms large-scale blooms in high-latitude oceans with a significant ecological and biogeochemical impact [5,6]. *E*. *huxleyi* displays a dimorphic haplodiplontic life cycle [7,8]. Diploid cells are typically covered with calcareous scales (coccoliths) and dominate natural blooms [9]. However, some strains lack coccoliths after extended periods in culture and are denoted ‘naked’. Haploid cells are also devoided of coccoliths, but are biflagellate and their cell membrane is coated with thin organic scales. Therefore, 1N cells have been denoted scale-bearing swarmers or ‘S-cells’ [7]. Both 2N and 1N cells can grow independently by mitosis and likely interconnect through sex and meiosis, although sexual reproduction has never been observed in *E*. *huxleyi*.

The evolutionary stability of haplodiplontic life-cycle strategies is often interpreted as adaptation to fluctuating environments, where each life phase is better fit to different niches (Valero 1994, Hughes and Otto 1999). Here differences between niches is meant in a broad sense, and may include both abiotic (e.g. seasonal) and biotic (e.g. predators, pathogens) factors. In agreement with this view, it has been shown that while 2N *E*. *huxleyi* cells are sensitive to specific *E*. *huxleyi* viruses (EhV) that drive the termination of natural blooms [10–13], 1N cells are resistant to EhV [14]. Moreover, the same studies showed that biflagellate cells can emerge during viral infections, suggesting the occurrence of meiosis and the production of resistant cells in response to EhV. This process was metaphorically coined the ‘Cheshire Cat’ (CC) escape strategy, whereby a life-phase switch provides an escape mechanism from EhV. This could alleviate the viral pressure on host cells and potentially select for maintenance of a biphasic life-cycle strategy over evolutionary timescales [14]. This hypothesis has recently received support from exploration of the gene repertoire of *E*. *huxleyi* genotypes isolated from nutrient-rich areas, where blooms and EhV infections regularly seem to retain a biphasic sexual life cycle. In contrast, *E*. *huxleyi* genotypes isolated from low-productivity areas, where blooms do not develop and EhV are undetected, tend to lack the flagellar genes that are typically expressed in 1N cells. Arguably, these results suggest that in populations experiencing low viral pressure and low environmental variability, life cycling is not advantageous, and *E*. *huxleyi* cells may lose the ability to undergo sexual reproduction and produce 1N cells [15].

Although life-cycle transitions during viral infection play a pivotal role in *E*. *huxleyi*, the cellular mechanism underlying the CC strategy remains unclear. Here, we used morphological, ploidy and gene-expression analyses of meiosis- and life-phase-specific gene markers to test whether virus-resistant cells are produced during infection or are instead selected from a background subpopulation after elimination of the numerically dominant calcified diploid cells. In parallel, we also examined the fate of an *E*. *huxleyi* strain that seems to be unable to form biflagellate cells and that is plausibly impaired in CC capabilities. We further investigated whether life-phase transitions are induced by diffusible chemical cues (infochemicals) accumulated during infection. Collectively, our study provides novel insights into complex host–virus interactions and morphological differentiation in unicellular eukaryotes.

## Results

### Dynamics of viral infection and recovery of resistant cells

**Fig 1:**
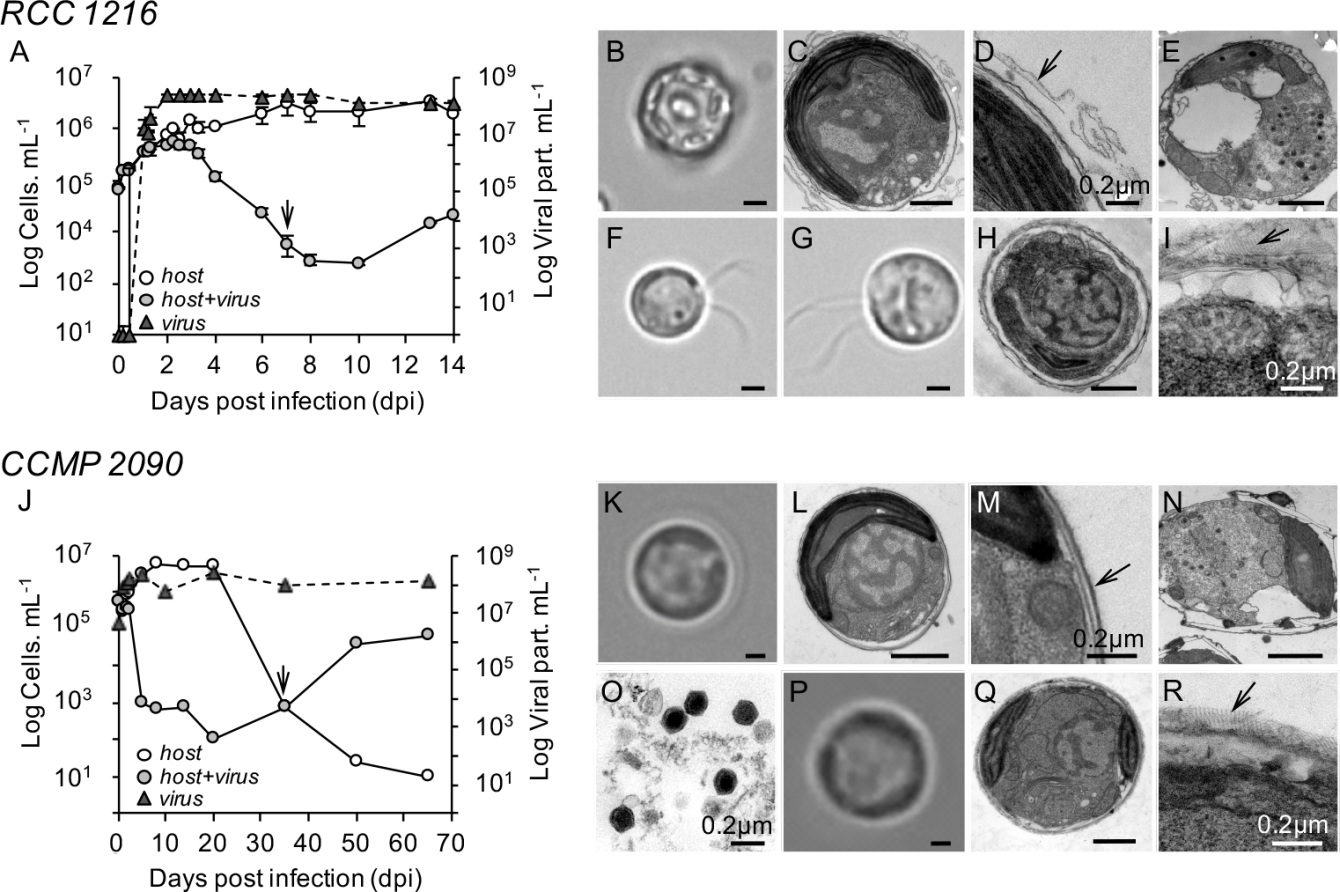
Host–virus dynamics and ultrastructural features of *E*. *huxleyi* cells. (A–I) Dynamic of growth infection and ultrastructural features of *E*. *huxleyi* RCC 1216, a 2N calcified strain. (A) Temporal dynamics of *E*. *huxleyi* during infection by EhV-201 compared to uninfected, control conditions. The arrow denotes the time (7 dpi) at which virus-resistant, biflagellate cells are first detected by light microscopy. (B–D) Light microscopy (LM) and transmission electron microscope (TEM) imagery of RCC 1216 cells. The close-up (D) highlights the presence of remnants of the organic matrix of coccoliths as indicate by an asterisk, but not organic scales. (E) TEM micrograph of an EhV-infected cell with multiple virions detected as dark electron-dense particles in the cytoplasm. (F) LM image of a haploid cell (RCC 1217), shown for comparison (see text for details). (G–I) LM and TEM images of a biflagellate cell isolated postinfection. The close-up (I) denotes the presence of organic scales (arrow) with patterns of fibrils bound to the cell membrane. (J–R) Dynamic of growth infection and ultrastructural features of *E*. *huxleyi* CCMP 2090, a 2N noncalcified strain. (J) Temporal dynamics of *E*. *huxleyi* during infection by EhV-201 as compared to uninfected, control conditions. The arrow denotes the time (35 dpi) at which virus-resistant nonmotile scale-bearing cells (nonmotile-S cell) were first detected by LM. (K–M) LM and TEM images of CCMP 2090 cells. (M) Arrow denotes the absence of organic scales bound to the cell membrane. (N) Cell infected by EhV, as described above in (E). (O) Close-up of EhV virions upon cell lysis. (P–R) LM and TEM images of nonmotile-S cells postinfection. The close-up (R) denotes the presence of organic body scales (arrow), as described in (I). The mean ± standard deviation of duplicate cultures is shown. The scale bars are 1 μm for all TEM images, except for images D, I, M, O and R in which the scale bar = 0.2 μm.

To investigate the molecular mechanisms underlying the CC strategy, we monitored the interplay between lytic virus EhV-201 [16] and two *E*. *huxleyi* strains: RCC 1216, a 2N calcified strain able to undergo sexual transitions and form biflagellate 1N cells [8]; and CCMP 2090, a 2N noncalcified strain, lacking essential flagellar genes and for which the production of 1N cells has never been recorded [15]. In the presence of EhV, both strains lysed to nearly undetectable levels. However, within variable time frames, a minor subpopulation of cells emerged and resumed growth in the presence of high EhV densities (Fig 1). During viral infection of RCC 1216 (Fig 1A–1I), there was a transient rise in noncalcified cells (low side-scattering subpopulation by flow cytometry) between 2 and 4 days postinfection (dpi). These cells comprised up to 35% of the total *E*. *huxleyi* population. Since at this stage, virtually all *E*. *huxleyi* cells were positive for the cell-death marker SYTOX-Green, this noncalcified population was essentially composed of dying cells which shed their coccoliths due to EhV infection (S1A Fig). However, at 7 dpi, we detected by light microscopy the presence of motile noncalcified biflagellate cells, either individually or in small motile groups of 3–6 cells. At this stage, we estimated that the motile fraction of cells represented ~0.05% of the maximal cell abundance at 2 dpi. Subsequent electron microscopy analyses revealed that the motile cells have thin organic scales with radiating patterns of fibrils (Fig 1G–1I), as is typical for *E*. *huxleyi* 1N cells [7] (Fig 1F). In the parallel assessement of strain CCMP 2090, we also detected the recovery of a new subpopulation, but it evolved over longer time scales of ~35 dpi as compared to 7 dpi in RCC 1216 (Fig 1J). The new emerging cells lacked flagella but had thin organic scales like 1N cells, as detected by electron microscopy (Fig 1P–1R). We termed these cells derived from CCMP 2090 nonmotile scaled cells (nonmotile-S cells).

### Gene-expression profiling during the course of infection

To assess whether the formation of biflagellate and nonmotile-S cells was driven by meiosis, we used qRT-PCR to monitor the expression of a core set of meiosis-associated genes (i.e., two *SPO11* variant genes, *DMC1*, *HOP1*, *MER3*, *MND1*, *MSH5* [17,18], see S1 Table and S2 Fig) together with a set of genes reported to be specific to 1N cells [15,19]. The latter, herein globally termed S-cell genes (S1 Table), included four flagellum-associated genes (*FLAG 4*, *5*, *8* and *11*), two phototropins (*PHOTO1*, *PHOTO*2), one *MYB* transcription factor and one histone *H2A*. RCC 1216 was examined at high temporal resolution to provide a comparative basis to our previous observations [14].

**Fig 2:**
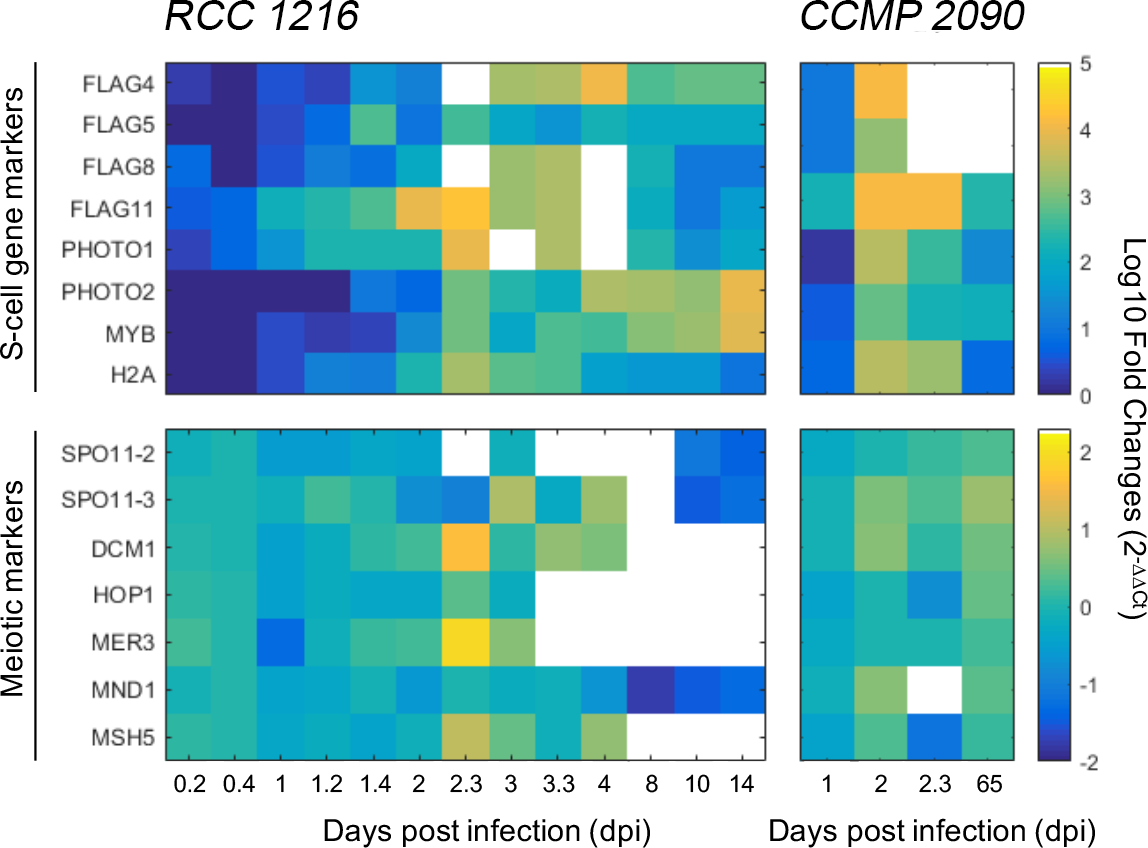
Gene-expression dynamics of meiosis- and life-phase-specific genes during EhV infection. Detailed expression profiles of profiles of meiosis- and S-cell-specific for *E*. *huxleyi* RCC 1216 and CCMP 2090 were performed by qPCR over the course of infection and are presented as heatmaps. The results are presented as Log_10_ fold-change (2^-ΔΔCt^) relative to control, uninfected cultures over time to enable of a clear visualization of the expression data encompassing a large range of variability. Blank heatmap cells represent time points with undetected gene expression levels. Gene expression was undetected for days 6 and 7 and therefore this time period was removed from the heatmap (gene expression data is available in S2 Table). The mean values of duplicate cultures are shown.

Coordinated upregulation of all S-cell gene markers (10^2^- to 10^4^-fold) was detected in both *E*. *huxleyi* strains over the course of EhV infection (Fig 2, S2 Table). Detailed analyses of RCC 1216 revealed upregulation of *FLAG11* and *PHOTO1* within 24 h of infection, followed by the upregulation of the remaining S-cell genes at 2 dpi. All S-cell gene markers remained above control levels until the end along with biflagellate cells growth. The expression levels of meiotic markers was lower than that of S-cell markers (often below noninfected control cultures) and markedly irregular over time (up/downregulation). The only clear exceptions were *HOP1* and *MER3* that showed a nearly 2-fold increase in expression at 2 dpi relative to control cells, concomitant with cell-growth arrest and onset of the lytic phase. The same general trend was also detected for CCMP 2090, where S-cell genes were markedly upregulated during infection, whereas meiotic genes showed lower or no variability relative to control cells. We note that *FLAG5* and *PHOTO1* genes were not detected in CCMP 2090, likely due to their absence from the genome of this strain [15].

In addition, we used the same set of S-cell and meiosis markers to assess whether the production of biflagellate cells in RCC 1216 could be triggered in response to diffusible signals (infochemicals) produced during infection (S3A Fig). Therefore, diploid RCC 1216 cultures were exposed at 50% vol/vol to virus-free lysates (VFL), a conditioning medium derived from infected cultures. VFL was harvested at 4 h, 24 h, 48 h and 72 h postinfection. We also used UV-inactivated EhV virions (virus-to-host ratio = 5) to examine the cellular response to potential virus-borne elicitors. However, gene-expression analyses did not reveal any noticeable gene upregulation after 4 h or 24 h of exposure to VFL as compared to typical EhV infections under any of these conditions (S3B Fig). Furthermore, the emergence of biflagellate cells could not be detected by light microscopy during the weeks following each treatment.

### Genome size and ploidy level analyses

**Fig 3:**
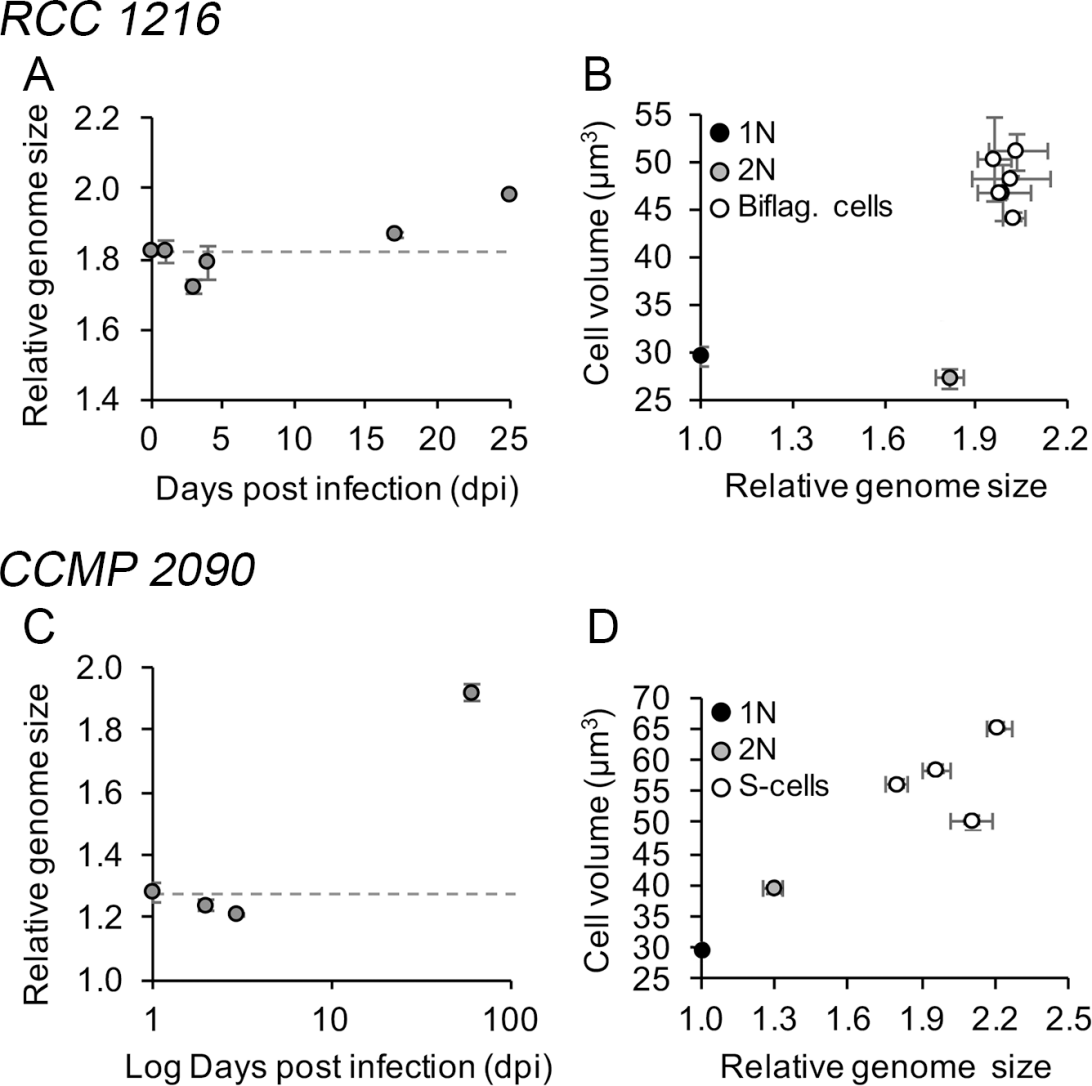
Genome-size analysis of *E*. *huxleyi* cells during EhV infection and recovery. Genome-size analysis represents the cells' DNA content as measured by the staining intensity of isolated nuclei in the G1 phase with the fluorescent probe SYBR-Green and assessed by flow cytometry (see methods). (A) Temporal variation of the average relative genome size of cells during EhV infection. (B) Relative genome size and cellular biovolume (μm^3^) of RCC 1216 (2N) and five representative biflagellate clones postinfection. We note that the cell biovolume of RCC 1216 was determined with cells treated with 1 μM EDTA to dissolve coccoliths and provide precise measurements of actual cell size. (C,D) as in (A,B) but using noncalcified *E*. *huxleyi* strain CCMP 2090, 2N. All of the data presented for relative genome size were normalized to the haploid (1N) genome of strain RCC 1217. The mean ± standard deviation of duplicate cultures is shown.

To further assess whether meiosis is occurring during infection, we examined the variations in relative genome size (RGS) of infected cells as compared to control cells (Fig 3). This was done by measuring the nuclear DNA content of cells by flow cytometry (S4 Fig) relative to the 1N strain RCC 1217 that was derived from RCC 1216 [8]. RCC 1216 cells possessed an average RGS of ~1.8xN, similar to previous reports [8], whereas diploid CCMP 2090 cells possessed an average RGS of ~1.3xN. In both RCC 1216 and CCMP 2090, the nuclear DNA content remained nearly invariable during the first 4 dpi (Fig 3A and 3C). However, RGS levels during cell recovery revealed higher values than the original parental diploid cells. Biflagellate cell populations displayed ~2xN RGS levels, representing an average ~10% increase relative to RCC 1216 (Fig 3A), whereas nonmotile-S cell populations displayed ~1.9xN RGS levels, representing an average ~60% increase relative to CCMP 2090 (Fig 3C).

To validate these results, we isolated representative single biflagellate and nonmotile-S cells. RGS levels of five independent biflagellate clones (LC4A, LC4F, LC4G, LC4I, LC4J) were consistently 2xN (Fig 3B), as detected for the total recovering populations. In contrast, the four nonmotile-S cell clones analyzed displayed variable RGS levels ranging from 1.8xN to 2.2xN, representing increments of ~40% to ~70% relative to CCMP 2090 (Fig 3D). Furthermore, both biflagellate and nonmotile-S cells were invariably larger (spherical cell volume) than the parental cell lines, by >50% and >25%, respectively (Fig 3B and 3D). Confocal microscopy observations did not reveal any irregular structural changes in the nuclei and cells were characterized by single nuclei, like the parental cell lines (S5 Fig).

In a complementary approach, we used the microsatellite marker P02F11 [20] to assess the ploidy level of resistant cells. This analysis revealed that all of the biflagellate clones are heterozygous, displaying doubled allele bands for two loci like the 2N RCC 1216 cells, and in contrast to the single bands detected in 1N RCC 1217 cells (S6 Fig). This indicates that the biflagellate cells have two chromosomal copies, as expected in 2N organisms.

### Growth profiles of recovered biflagellate and nonmotile-S cells

**Fig 4:**
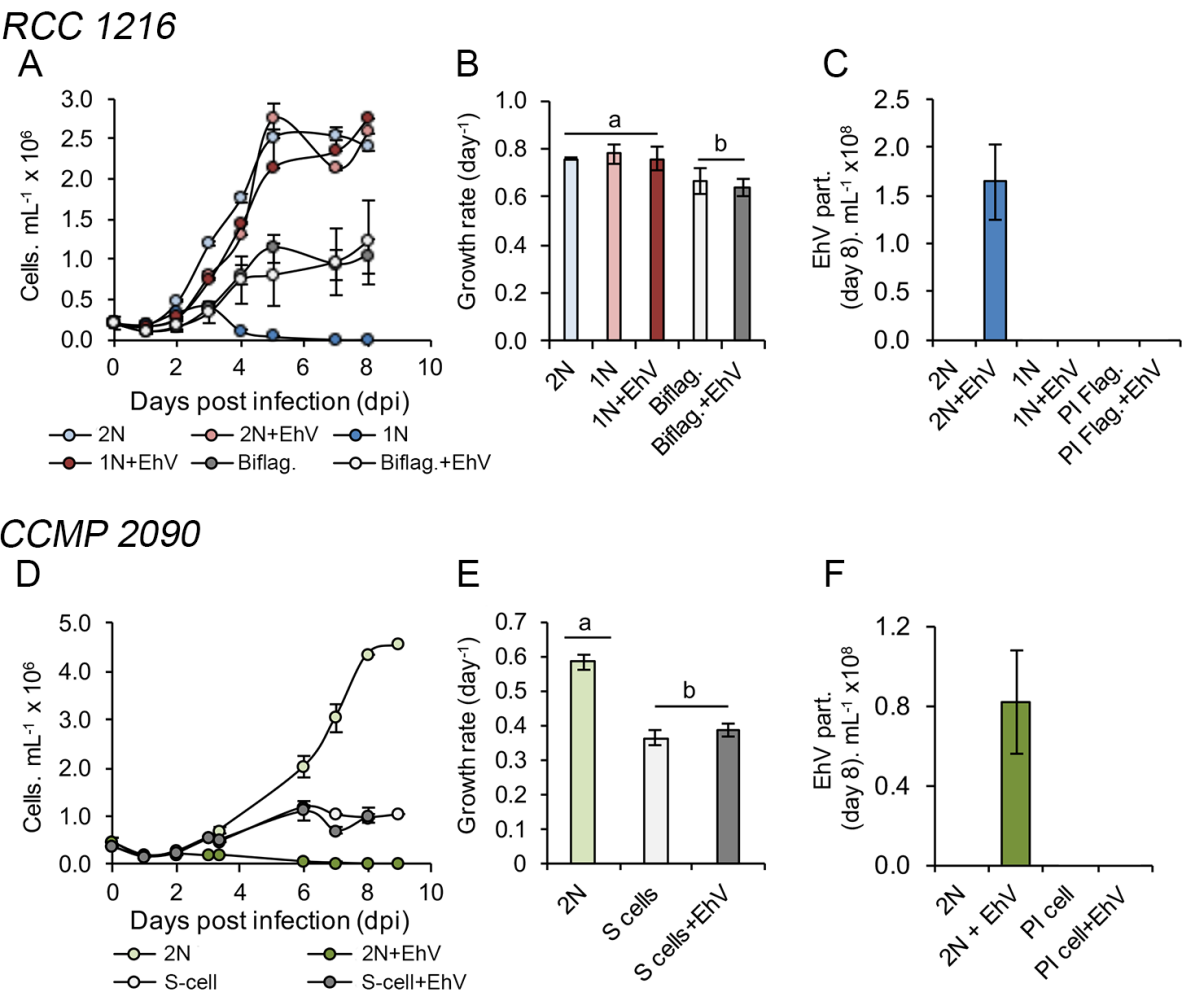
Growth dynamics and susceptibility to infection of biflagellate and nonmotile-S cells isolated after EhV infection. (A) Growth dynamics of a representative biflagellate clone (PI Flag.) relative to RCC 1216 (2N) and RCC 1217 (1N) in controls and during viral infection by EhV-201. (B) Growth rate and (C) EhV production as determined by flow cytometry. (D) Growth dynamics of representative nonmotile-S cells (PI Flag.) relative to CCMP 2090 (2N) in controls and in the presence of EhV-201. (E) Growth rate and (F) EhV virion production as determined by flow cytometry. The symbols a and b shown in (B) and (E) distinguish treatments groups that are identical from those that are different, respectively, following statistical analyses (*t*-test). *E*. *huxleyi* RCC 1216, 2N calcified strain was the parental strain for clonal isolation in (A–C), and *E*. *huxleyi* CCMP 2090, 2N calcified strain in (D–F). The mean ± standard deviation of triplicate cultures is shown.

To provide a functional characterization of biflagellate and nonmotile-S cells, we conducted growth assays of all isolated clones and examined their susceptibility to viral infection, in comparison to the parental strain RCC 1216, RCC 1217 and CCMP 2090 (Fig 4). Both biflagellate and nonmotile-S cells exhibited significantly lower growth rates and carrying capacities (average cell density during stationary phase) than each parental cell line. Fitness reduction in the five biflagellate cell lines was diagnosed by an average ~15% decline in growth rate (*t*-test, *P* < 0.05) and ~55% decline in carrying capacity (*t*-test, *P* < 0.01) relative to RCC 1216 (Fig 4B). In nonmotile-S cells, fitness decline was more severe, with growth rates declining ~30% (*t*-test, *P* < 0.01) and carrying capacities declining ~75% (*t*-test, *P* < 0.01) relative to CCMP 2090 (Fig 4E). Importantly, both biflagellate and nonmotile-S clones were resistant to EhV infection, i.e., their growth in the presence of EhV was similar to that under control conditions and the production of new viral particles was not detectable by flow cytometry (Fig 4C and 4F).

## Discussion

### Life-phase transition is triggered in response to viral infection

The CC strategy originally described *E*. *huxleyi's* ability to escape from EhV by alternating from a 2N virus-sensitive phase to a morphologically distinct 1N virus-resilient phase [14]. Here, we reassessed the CC strategy to further understand the mechanisms mediating the life-cycle shift in *E*. *huxleyi* in response to EhV. For RCC 1216 (2N), we detected the emergence of biflagellate cells that were resistant to viral infection following the lysis of calcified 2N cells (Fig 1), recapitulating earlier observations [14]. In contrast, the response of 2N CCMP 2090 cells to EhV was unprecedented. These cells, which are unable to form motile cells and may lack the ability to undergo sexual transitions [15], produced nonmotile-S cells during viral infection that bore organic scales similar to 1N cells. This result indicated that *E*. *huxleyi* cells lacking flagellar genes [15] can still undergo life-phase transitions and may retain a sexual life cycle. Additional laboratory and field work, possibly making use of population genomic approaches [21], is required to further understand the complexity of *E*. *huxleyi*'s life cycle in its natural habitats, including in oligotrophic systems where cells displaying genomic erosion of flagellar genes seem to predominate.

Originally, the CC model stated that virus-resistant cells (haploid) are produced through meiosis in response to EhV infection [9,14]. However, there is some doubt as to whether instead of a sexual transition, the CC strategy might involve a selection process, where low background levels of 1N cells take over after lysis of the virus-susceptible 2N cells. Our detection of marked and rapid overexpression of S-cell genes during infection (Fig 2) provides molecular support for the original view that a life-phase transition is triggered during infection. However, based on our population-level analyses, the fraction of the population undergoing this life-phase transition remains unclear. Further single-cell approaches are required to quantify this rare subpopulation during infection [22]. Moreover, we did not detect any phenotypic response to diffusible cues accumulated during infection, suggesting that a direct host–virus interaction may be required to trigger the production of resistant cells (S3 Fig).

### The CC strategy is decoupled from meiosis

Although a life-phase transition seemed to be triggered during infection in the two tested *E*. *huxleyi* strains, the increment in RGS levels in cells recovered after infection indicated that meiosis is probably not involved in the process. In the case of biflagellate cells, both RGS measurements and microsatellite analyses indicated that these cells are 2N (Fig 3, S6 Fig). However, we did note a mismatch in RGS between biflagellate cells and the parental cell line RCC 1216, the first being 2xN and the second 1.8xN. The nature of this ~10% discrepancy requires clarification through further cytogenetic and genomic analyses but it might have resulted from a bias in our ploidy-level analyses by flow cytometry as a result of differential condensation levels of the DNA and staining efficiencies in the two cell types, as has been documented in other systems [23]. This being the case, the biflagellate cells are regular 2N cells, produced via a phenotypic-switch mechanism that is independent of the sexual cycle. Such decoupling between life-phase phenotype and ploidy level resembles apomictic life cycles observed in haplodiplontic plants and algae [24,25], which can be triggered under stress conditions [26]. Apomixis can involve either apospory with the formation of a 2N gametophyte (typically the 1N phenotype) without meiosis, or apogamy with the formation of a 1N sporophyte (typically the 2N phenotype) without syngamy. Some evidence suggests that these types of processes can also occur in other noncalcified prymnesiophytes related to *E*. *huxleyi* [27–29]. Thus, it is plausible that phenotype remodeling through apospory, putatively mediated through genetic or epigenetic mechanisms [30–32], is at the basis of the formation of biflagellate diploid cells in response to EhV-mediated stress.

In the case of nonmotile-S cells, the explanation is less straightforward because these cells exhibit a variable range of aneuploid genomes considerably larger than CCMP 2090 (Fig 3). Thus, it is possible that major genomic rearrangements, including the duplication of chromosomal parts and disruption of other sections, led to differential regulation of gene expression and subsequent production of modified phenotypes. This could have occurred during viral infection [33,34] or be a host specific response to EhV. Aneuploidization involving chromosomal duplication (or partial duplication) has well-documented roles in adaptation conferring for example fitness advantages under a variety of abiotic (e.g. temperature, nutrients) and biotic stress conditions [35–37], including resistance to viruses as recently reported in a marine picoeukaryote [38]. However, given that nonmotile-S cells also exhibited morphological phenotypes resembling haploid cells (i.e., presence of organic scales), we argue that a similar aposporic mechanism was also involved in the production of these cells in response to EhV.

Given that meiosis did not appear to underlie the production of biflagellate and nonmotile-S cells, the role of meiotic gene transiently detected during infection (Fig 2) is unclear. Meiosis is a necessary part of sexual reproduction and a core set of genes involved in DNA double-strand break formation and crossover regulation seem to be conserved and to be fairly specific across eukaryotic lineages [17,18,39]. However, it has been shown that some meiosis-related genes can also play a role in other recombination mechanisms [39–41]. During EhV infection, while host cells undergo major cellular and metabolic reprogramming, pathways related to DNA repair are upregulated [42,43]. Moreover, we could also detect the expression of meiotic genes under control conditions, implying an alternate role for these genes in other cellular processes that could have been further enhanced during infection.

### Viral resistance and growth of biflagellate and nonmotile-S cells

Cell-growth assessment of recovered biflagellate and nonmotile-S clones confirmed a stable resistance phenotype to EhV-201 (Fig 4). During a parallel test, we found that both cell types were also resistance to diverse EhV strains (EhV-86, EhV-163, EhV-ice 01 [16,44]), which suggests the existence of a generic, albeit unknown resistance mechanism against EhV that is common to all cells expressing a phenotype resembling the haploid cell. Further analyses of the processes of EhV adsorption onto host cells and the role of the organic scales or cell-surface properties of resistant cell lines may provide new insights into the mechanisms of viral resistance.

Concomitantly, both biflagellate and nonmotile-S cells showed compromised growth fitness as compared to the parental cell lines under control conditions, i.e., decreased growth rate and carrying capacity (Fig 4). Tradeoff costs are often detected in bacterial and eukaryotic cells after the acquisition of resistance to viruses [45–50]. Here, it is possible that a fitness decline resulted from the increased cell volumes and consequent decline in cell surface-to-volume ratio, which often leads to decreasing nutrient-uptake rates and growth [51]. In nonmotile-S cells that showed higher levels of fitness decline, a decrease in nutrient-uptake rates may have been associated with additional physiological costs for DNA biosynthesis or other genomic destabilization following aneuploidization [35,52].

Recent analyses of control 1N RCC 1217 cells detected minute amounts of viral glycosphingolipids and EhV transcripts, suggesting a possible mode of persistence of EhV within haploid cells [53,54]. To determine whether this might also be the case in biflagellate and nonmotile-S strains, we extracted both DNA and RNA from various clones and screened for EhV-specific gene markers. However, all of the results were negative, indicating that none of the resistant strains carry any form of EhV.

The suite of isolated biflagellate and nonmotile-S cell cultures will provide a powerful tool for future cellular and comparative omics analyses to dissect the cellular mechanism enabling morphological remodeling and viral resistance in *E*. *huxleyi*.

### Conclusions

**Fig 5:**
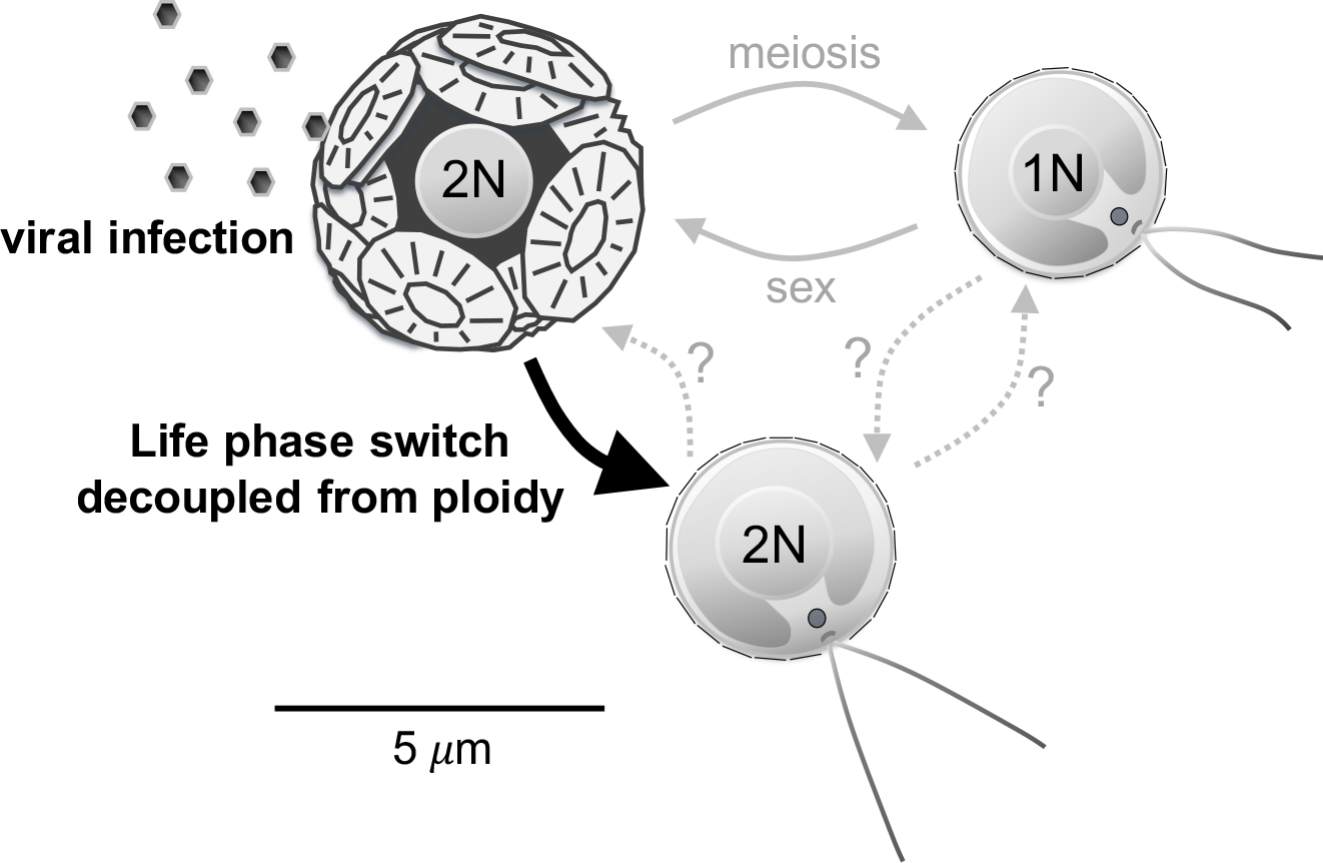
Schematic representation of the ‘Cheshire Cat’ mechanism in *E*. *huxleyi* in response to viral infection. As currently described, the haplodiplontic life cycle of *E*. *huxleyi* comprises a calcified diploid and noncalcified scale-bearing biflagellate haploid stage that is resistant to specific viruses (EhV). Both diploid and haploid cells likely interconnect through meiosis and syngamy. Here, however, we show that in response to infection by EhV, *E*. *huxleyi* can produce diploid biflagellate and scale-bearing cells that are resistant to infection as indicated by the black arrow. This mechanism seems to be decoupled from the regular sexual cycle and to enable *E*. *huxleyi* cells to rapidly respond to and escape EhV infection. The fate of the diploid biflagellates is unknown, but they may revert back to the calcified state or undergo meiosis to produce regular haploid cells.

Our results provide novel evidence for a CC model in which *E*. *huxleyi* cells' ability to escape viral attack through life-phase change is decoupled from the sexual cycle; this stands in contrast to the original CC scheme [14] (Fig 5). This process seems to be triggered in a small fraction of cells during infection by EhV and to enable the production of diploid (or aneuploid) cells that display phenotypes resembling haploid cells and that are resistant to EhV. The morphological and genome-size properties of both biflagellate and nonmotile-S cells have been stable in culture for the last 1.5 years of isolation. However, it is plausible that these cells constitute an intermediary state produced under stressful conditions and that they are capable of reverting back to the original calcified nonmotile state, or instead undergoing meiosis to resume life-cycle progression (Fig 5). This extended capability of decoupling phenotype from ploidy level may improve the adaptability of these microbial cells to the highly fluctuating stressful conditions at sea and enhance survival rates during the interplay with EhV during bloom events.

## Materials and methods

### Cell culturing, viral infection and analytical measurements

Replicate cultures of *E*. *huxleyi* RCC1216 (calcifying, diploid; previously referred to as strain TQ26 [8]) and CCMP 2090 (noncalcified, diploid; equivalent to strain CCMP 1516 for which genomic information is available [55]) were grown in seawater-based K/2 medium [56] and infected with the lytic viral strain EhV-201 [16] at a virus-to-host ratio of 0.2 (initial 10^5^ cell mL^-1^). Noninfected cultures were used as controls. The haploid *E*. *huxleyi* strains RCC 1217 (isolated from RCC 1216 after sporadic diploid-to-haploid transitions, [8]) was grown under identical conditions and used for comparative assays.

Cells and EhV were enumerated by flow cytometry (Eclipse, iCyt equipped with 488-nm solid-state air-cooled laser and standard filter setup) [57]. Algal cells were differentiated based on chlorophyll autofluorescence and side-scatter signatures, enabling the segregation of calcified from noncalcified cells (higher and lower side scatter, respectively) [14]. Algal cell death was determined with 1 μM SYTOX Green (Invitrogen) by flow cytometry [58]. The diameter of the cells was assessed with a Multisizer 4 Coulter counter (Beckman Coulter). Relative average nuclear DNA content (RGS) was monitored by flow cytometry (LSR, BD Biosciences) using extracted cell nuclei labeled with the fluorochrome SYBR Green [8] and the 1N RCC 1217 as an internal standard for data normalization.

### Microscopy

Light microscopy was performed using a differential-phase contrast setup at x100 magnification (Olympus, Japan). Electron transmission microscopy preparation was performed as described in Schatz and Shemi [59]. *E*. *huxleyi* cell nuclei and chloroplasts were observed with a confocal microscope (Eclipse Ti-E Inverted microscope, Nikon, Japan) using cells fixed with 1% formaldehyde for 2 h at 4°C and stained with 5 μg mL^-1^ SYTO13 Green (Molecular Probes) for 10 min.

### Microsatellite band-pattern analysis

Genomic DNA was extracted from cell pellets (~10^6^ cells) from RCC 1216, RCC 1217 and all biflagellate clones isolated in this study using a standard phenol–chloroform extraction method. The microsatellite marker P02F11 was amplified by PCR [20] and the products separated by electrophoresis using a Criterion TGX Any kD precast gels with Tris-borate buffer at 50 V for ~3.5 h. The size of the amplified products was determined using a standardized 100 bp DNA ladder (Promega).

### Gene markers: Annotation and phylogenetic analyses

Meiotic genes (S1 Table) were manually defined and aligned as described in Feldmesser et al. [60]. Briefly, the choice of target genes was based on a list of core meiosis genes from other protists (e.g. [17,18]) and *Arabidopsis* previously published and deposited at the National Center for Biotechnology Information (NCBI) and the Joint Genome Institute (JGI). All hits were analyzed for transcript evidence (ESTs) or gene models. A gene model was then built manually based on the existing transcripts, models, and BLAST results. Whenever transcriptome information was available [42] it was used to improve the manual gene models. After the definition and translation of *E*. *huxleyi* genes, the protein sequence was used for searches against the protein collections of NCBI. Multiple sequence alignments and phylogenetic analyses were performed using maximum likelihood with Mega (version 7.0.16) (S2 Fig). The manually curated genes from *E*. *huxleyi* were deposited in GenBank (KY224381–KY224389) and are detailed in S1 Table. S-cell genes were all derived from previous studies as listed in S1 Table.

### RNA isolation and qRT-PCR analysis

250-mL cultures collected by centrifugation (8000*g*, 4°C, 10 min) at each time point. RNA was isolated with the RNeasy Plant Mini Kit (Qiagen) according to the manufacturer’s instructions. Following DNAse treatment (Turbo DNAse, Ambion), the RNA was reverse-transcribed to cDNA with the ThermoScript RT-PCR system (Invitrogen). Transcript abundance was determined with the Platinum SYBR Green qPCR SuperMix-UDG with ROX (Invitrogen). Primers are listed in S1 Table. All of the reactions were performed on StepOnePlus real-time PCR Systems (Applied Biosystems) as follows: 50°C for 2 min, 95°C for 2 min, 40 cycles of 95°C for 15 s, 60°C for 30 s. Relative gene expression of each gene was calculated using the 2^-ΔΔCt^ method [61] against control uninfected samples per time point.

To test whether the RCC 1216 and RCC 1217 strains and the new biflagellate clonal strains contained intracellular EhV, we subjected 300 ng of host DNA or RNA to qPCR for several viral genes: viral major capsid protein [16] and viral serine palmitoyl transferase [22].

### Bioassays with conditioning medium and infection-derived cues

The effect of chemical signals that might trigger the production of virus-immune cells was tested using conditioned medium derived from infection. Briefly, *E*. *huxleyi* RCC 1216 cultures (10 L) were infected with EhV (virus-to-host ratio of 0.2), and 200-mL subsamples were collected at 4 h, 24 h, 48 h and 72 h postinfection and sequentially filtered through a 0.45-μm filter and 300 kDa tangential-flow filtration system (PALL) to remove cells and EhV particles. The filtrate (VFL, see scheme in S3 Fig) from each time point was then added (50:50 vol/vol) to exponentially growing RCC 1216 cultures at 5 x 10^5^ cell mL^-1^. Controls were diluted in the same proportion with fresh K/2 medium. Samples (200 mL) for microscopy and qRT-PCR analyses were collected at 4 h and 24 h after exposure to VFL. In addition, we tested the effect of EhV-derived components. A 50-fold concentrate of purified EhV virions [59] was exposed to 4000 μJ of UV light (UV Stratagene) to inhibit viral activity and added to cultures at a virus-to-host ratio of 5. EhV particles not exposed to UV were used as a positive control. Samples (200 mL) for microscopy and qRT-PCR analyses were collected at 4 h, 24 h and 48 h. In all of the assays, cells and viral enumeration as well as gene-expression analyses were assessed according to the procedures described above.

## Supporting information

S1 Fig Cell death and variation of noncalcified cells during EhV infection and control conditions. (A) Percentage of total, calcified and noncalcified cells positively labeled with the cell-death marker SYTOX-Green. (B) Noncalcified cells were detected as low side-scattered cells by flow cytometry. The arrow indicates the emergence of biflagellate cells postinfection (see text for details). The mean ± standard deviation of duplicate cultures is shown.
(TIF)

S2 Fig Molecular phylogenies of meiosis genes. Meiosis proteins for *SPO11-2*, *SPO11-3*, *DMC1*, *HOP1*, *MER3*, *MND1* and *MSH5* genes of *E*. *huxleyi* (red) were analyzed along with representative sequences of other organisms with defined gene sequences using Maximum Likelihood method. Among-site substitution rate heterogeneity was corrected using gamma-distributed substitution rate for invariant sites (G+I) and LG substitution model for amino acid substitutions. The bootstrap consensus tree was inferred from 500 replicates.
(TIF)

S3 Fig Effect of viral-derived infochemicals on life-phase transition. (A) Schematic representation of procedure to obtain and expose fresh cultures to conditioned medium from an infection. (B) Expression profiles of ‘motile-cell-specific’ and meiotic genes during VFL and UV-treated EhV experiments. Composite heat map represents the expression profiles (fold-change) of *E*. *huxleyi* RCC 1216 at 4 h and 24 h after UV treatment, and 4 h, 24 h and 48 h after EhV treatment. Control and EhV-infected cultures collected at 24 h or 48 h were used as negative and positive controls, respectively. Under all conditions, neither gene-expression analysis revealed noticeable gene upregulation as compared to typical EhV infections. The mean ± standard deviation of triplicate cultures is shown.
(TIF)

S4 Fig Flow cytometry histogram plots of the temporal variation of genome size of *E*. *huxleyi* cells during EhV infection. Panels on the left are for RCC 1216 and on the right for CCMP 2090. Measurements of relative genome size were collected during EhV infection at the time points shown in Fig 1. Haploid strain RCC 1217 (histogram) was used as an internal standard for data normalization. The mean ± standard deviation of duplicate cultures is shown.
(TIF)

S5 Fig Confocal microscopic imaging of *E*. *huxleyi* cells. From left to right: chloroplast, nuclei, and merged chloroplast, nuclei and phase-contrast microscopic imaging. (A–C) RCC 1216 2N calcified cells. (D-F) RCC 1217 1N cells. (G-I) Representative biflagellate cell derived from RCC 1216 after infection. (J-L) CCMP 2090, 2N noncalcified. (M-O) Representative nonmotile-S cell derived from CCMP 2090 after infection.
(TIF)

S6 Fig Microsatellite profiling of *E*. *huxleyi* cells. The microsatellite marker P02F11 [62] was used to analyze the ploidy level of five representative postinfection biflagellate clones. The 2N RCC 1216 and the 1N RCC 1217 (1N) were used as references. P02F11 amplifies two loci (A and B) that are heterozygous in diploid RCC 1216 and homozygous in haploid RCC 1217 cells.
(TIF)

S1 Table Target genes used in the study, putative function, sequence ID used for primer design (GS prefix denotes EST cluster from [22] and GenBank accession numbers of genes annotated in this study). (DOCX)

S2 Table Gene expression values (2^-ΔΔCt^) obtained by qPCR of S-cell and meiosis genes for RCC 1216 and CCMP 2090 during EhV infection used in the heatmaps presented in Fig 2. (CSV)
