# Supplementary figures and images for: Morphological switch to a resistant subpopulation in response to viral infection in the bloom-forming coccolithophore Emiliania huxleyi
Source: PLoS Pathog. 2017 Dec 15;13(12):e1006775. doi: 10.1371/journal.ppat.1006775 (PMC5756048; doi:10.1371/journal.ppat.1006775)

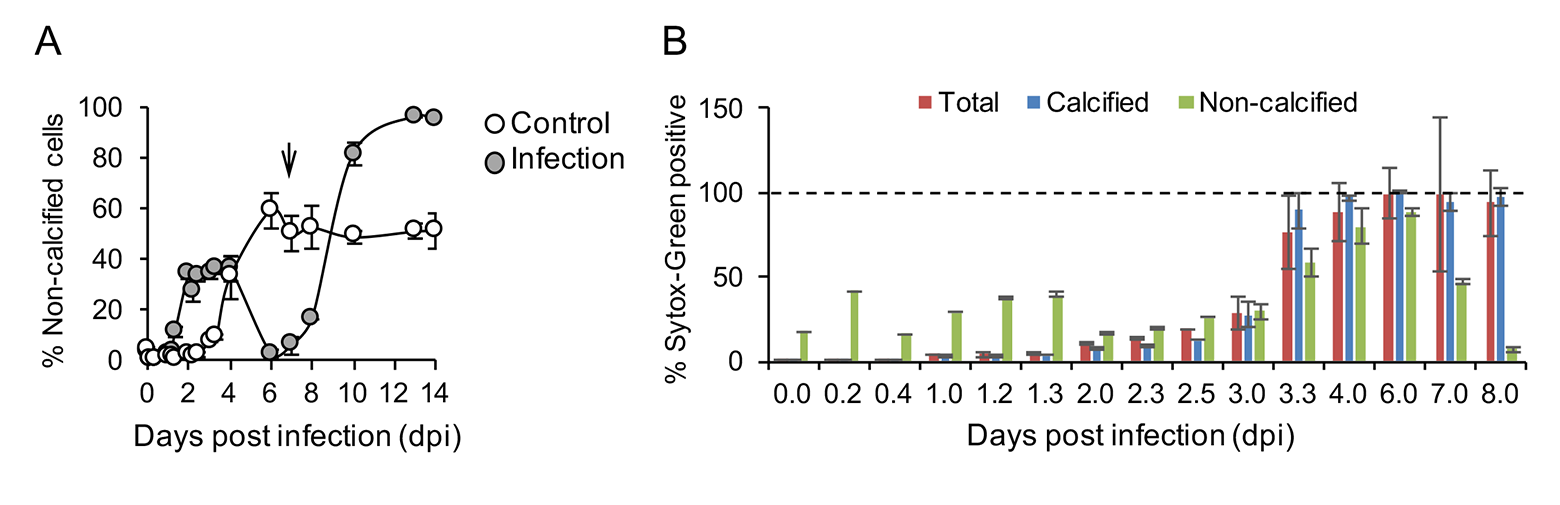

Supplement: S1 Fig — (A) Percentage of total, calcified and noncalcified cells positively labeled with the cell-death marker SYTOX-Green. (B) Noncalcified cells were detected as low side-scattered cells by flow cytometry. The arrow indicates the emergence of biflagellate cells postinfection (see text for details). The mean ± standard deviation of duplicate cultures is shown. (TIF) [file ppat.1006775.s001.tif]

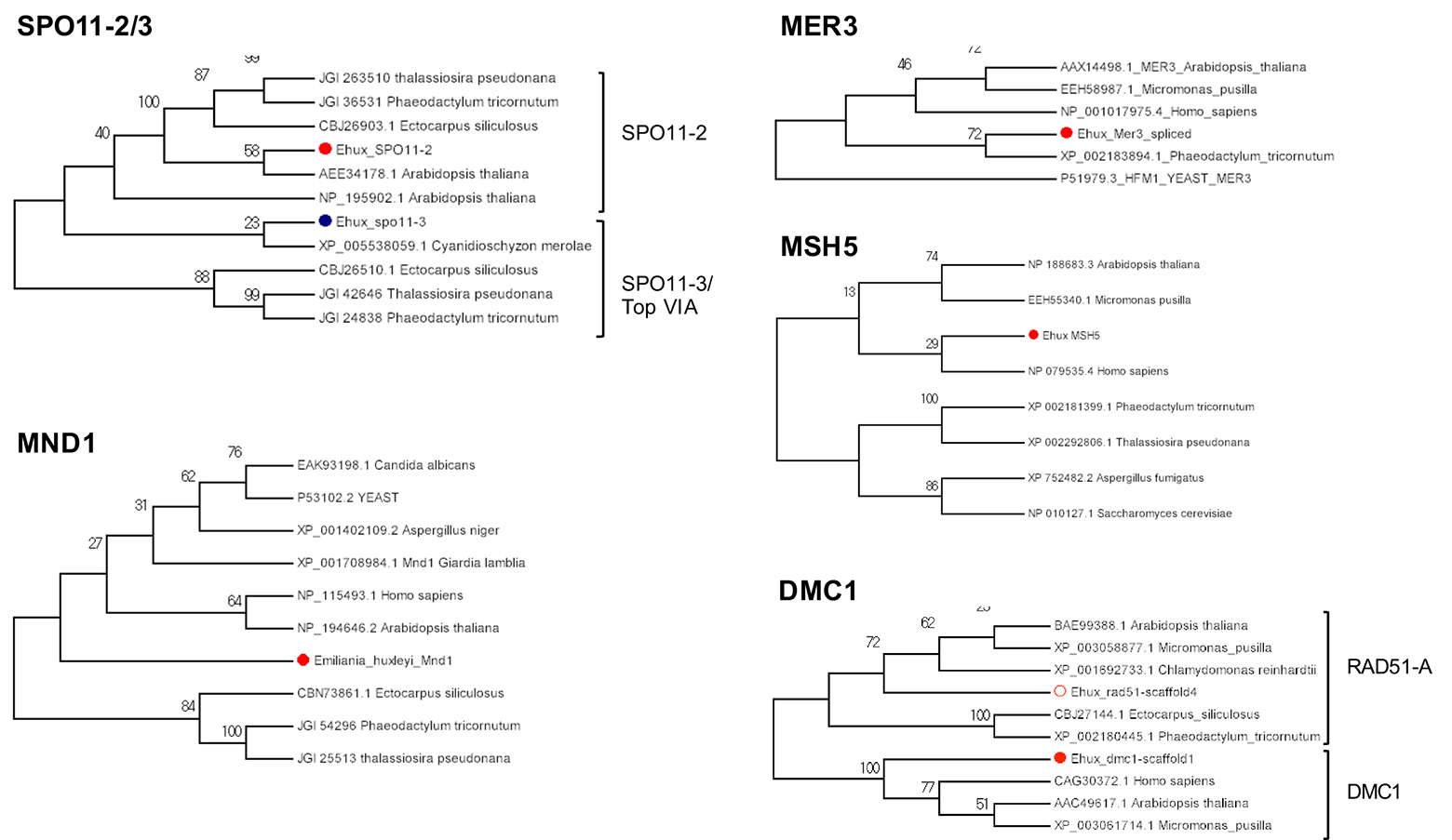

Supplement: S2 Fig — Meiosis proteins for SPO11-2, SPO11-3, DMC1, HOP1, MER3, MND1 and MSH5 genes of E. huxleyi (red) were analyzed along with representative sequences of other organisms with defined gene sequences using Maximum Likelihood method. Among-site substitution rate heterogeneity was corrected using gamma-distributed substitution rate for invariant sites (G+I) and LG substitution model for amino acid substitutions. The bootstrap consensus tree was inferred from 500 replicates. (TIF) [file ppat.1006775.s002.tif]

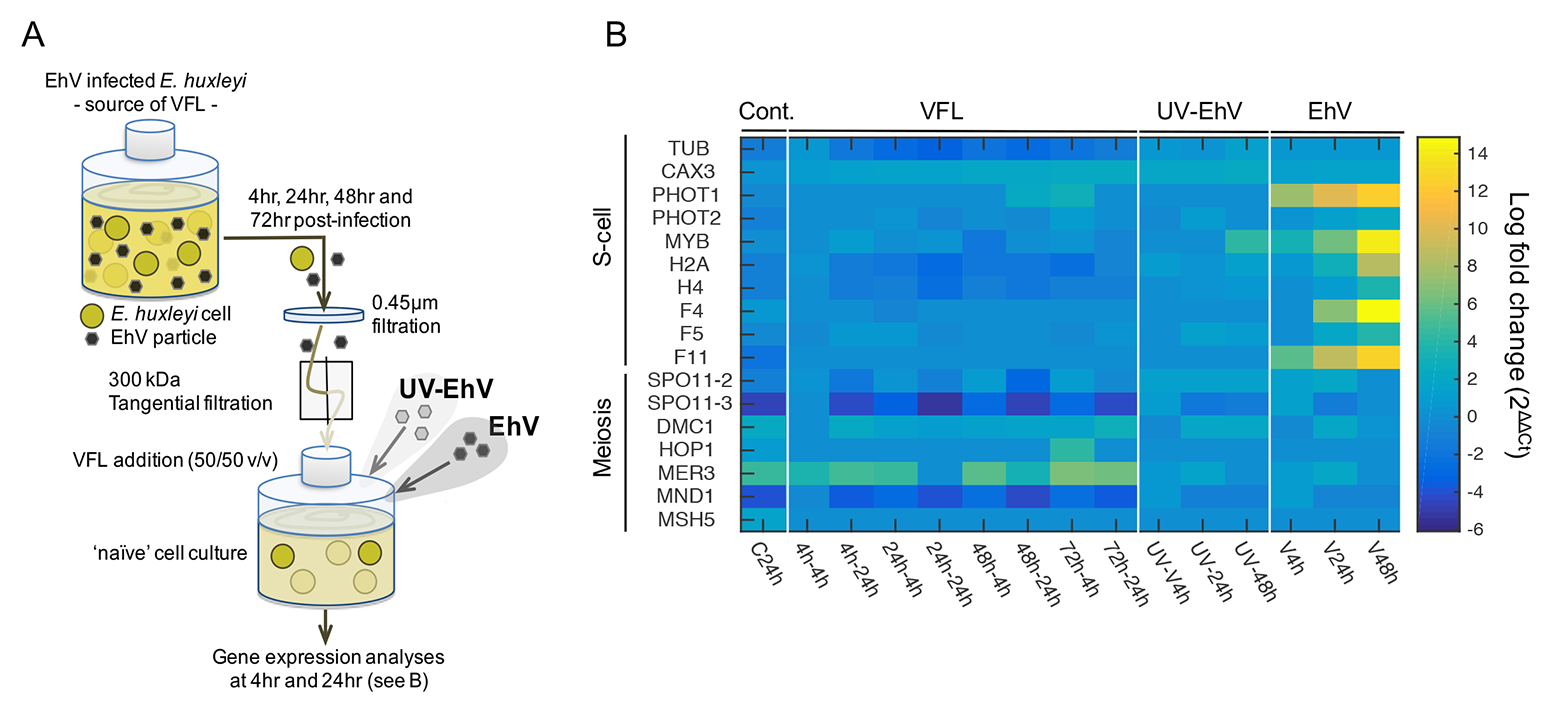

Supplement: S3 Fig — (A) Schematic representation of procedure to obtain and expose fresh cultures to conditioned medium from an infection. (B) Expression profiles of ‘motile-cell-specific’ and meiotic genes during VFL and UV-treated EhV experiments. Composite heat map represents the expression profiles (fold-change) of E. huxleyi RCC 1216 at 4 h and 24 h after UV treatment, and 4 h, 24 h and 48 h after EhV treatment. Control and EhV-infected cultures collected at 24 h or 48 h were used as negative and positive controls, respectively. Under all conditions, neither gene-expression analysis revealed noticeable gene upregulation as compared to typical EhV infections. The mean ± standard deviation of triplicate cultures is shown. (TIF) [file ppat.1006775.s003.tif]

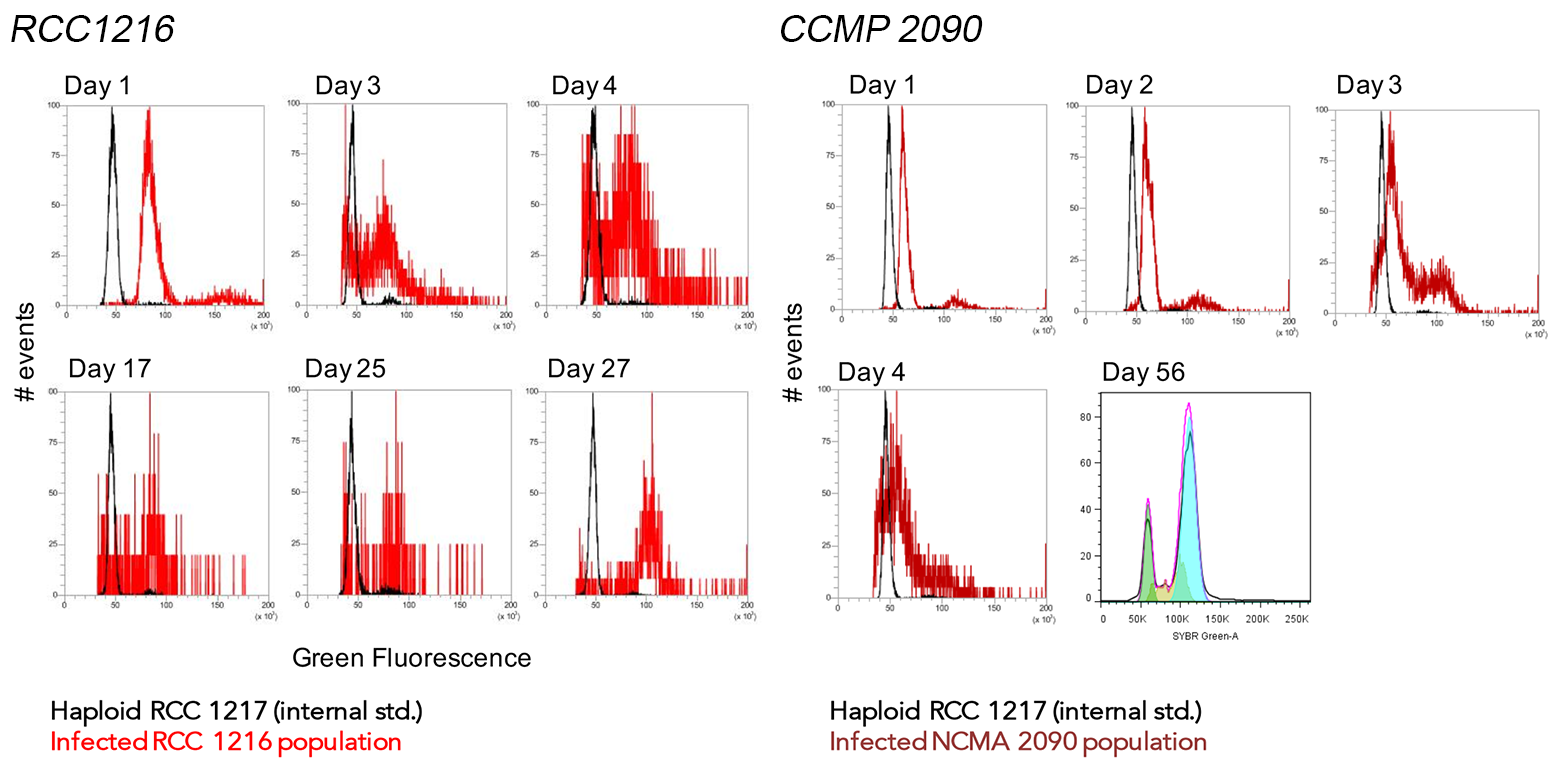

Supplement: S4 Fig — Panels on the left are for RCC 1216 and on the right for CCMP 2090. Measurements of relative genome size were collected during EhV infection at the time points shown in Fig 1. Haploid strain RCC 1217 (histogram) was used as an internal standard for data normalization. The mean ± standard deviation of duplicate cultures is shown. (TIF) [file ppat.1006775.s004.tif]

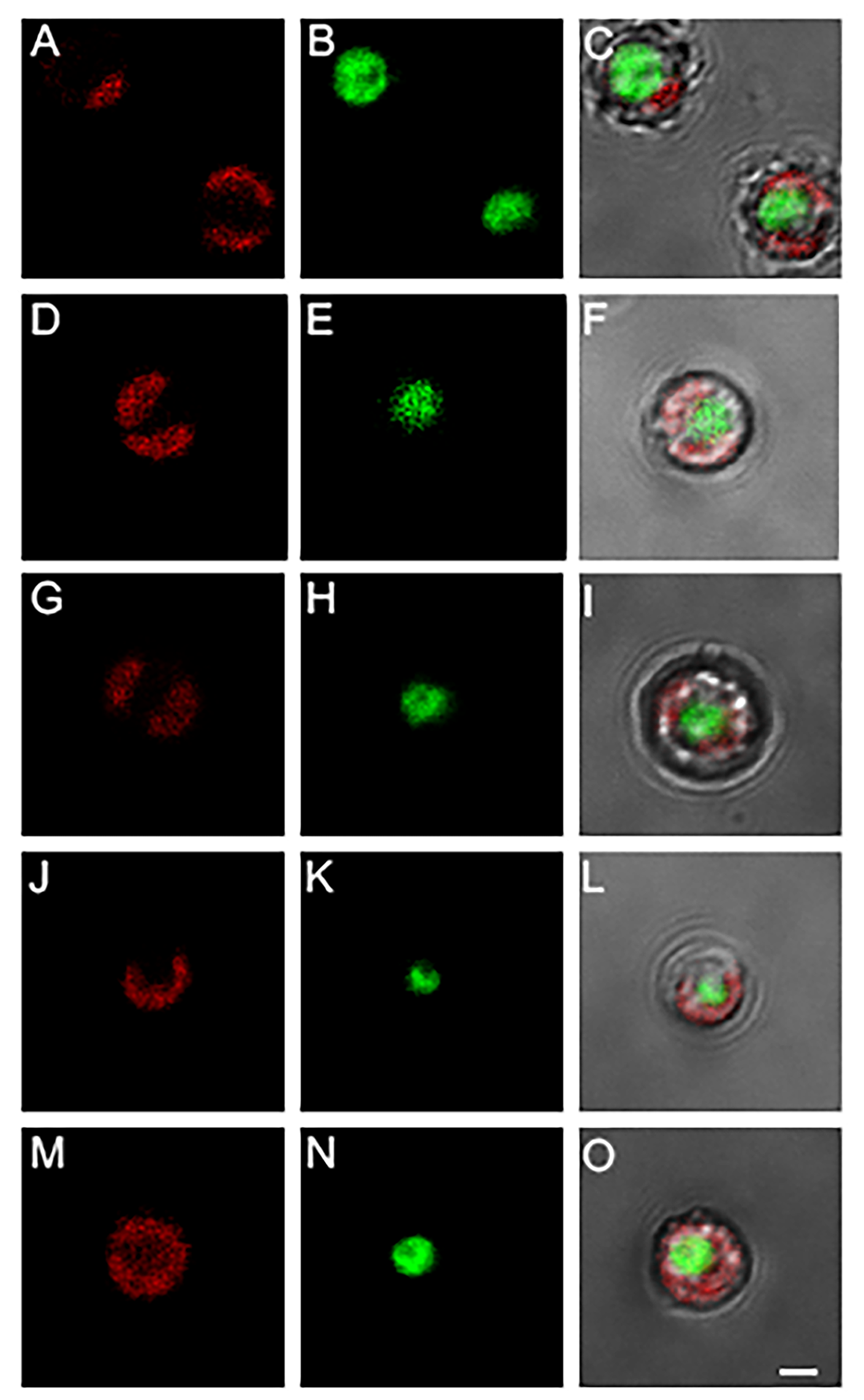

Supplement: S5 Fig — From left to right: chloroplast, nuclei, and merged chloroplast, nuclei and phase-contrast microscopic imaging. (A–C) RCC 1216 2N calcified cells. (D-F) RCC 1217 1N cells. (G-I) Representative biflagellate cell derived from RCC 1216 after infection. (J-L) CCMP 2090, 2N noncalcified. (M-O) Representative nonmotile-S cell derived from CCMP 2090 after infection. (TIF) [file ppat.1006775.s005.tif]

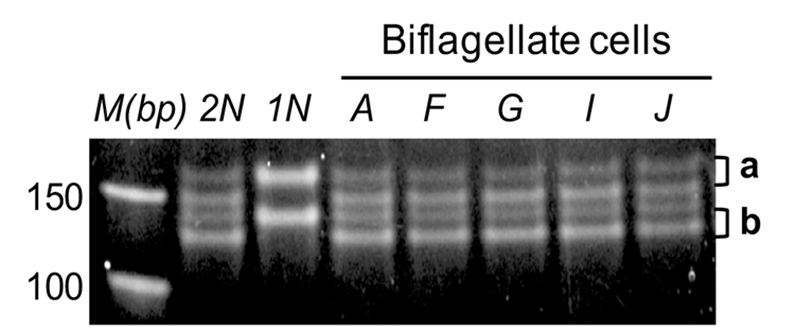

Supplement: S6 Fig — The microsatellite marker P02F11 [62] was used to analyze the ploidy level of five representative postinfection biflagellate clones. The 2N RCC 1216 and the 1N RCC 1217 (1N) were used as references. P02F11 amplifies two loci (A and B) that are heterozygous in diploid RCC 1216 and homozygous in haploid RCC 1217 cells. (TIF) [file ppat.1006775.s006.tif]
