# Supplementary material for: Morphological switch to a resistant subpopulation in response to viral infection in the bloom-forming coccolithophore Emiliania huxleyi
Source: PLoS Pathog. 2017 Dec 15;13(12):e1006775. doi: 10.1371/journal.ppat.1006775 (PMC5756048; doi:10.1371/journal.ppat.1006775)
Supplement: S1 Table — (DOCX) [file ppat.1006775.s007.docx]

**S1 Table.** Target genes used in the study, putative function, sequence ID used for primer design (GS prefix denotes EST cluster from [22] and GenBank accession numbers of genes annotated in this study).

| Gene | Function | Sequence Genbank ID | Primer name | Primer sequence (5'→3') | Reference |
| --- | --- | --- | --- | --- | --- |
| *SPO11-2* | Type II DNA topoisomerase VI subunit A (SPO11) homolog 2 | KY224381 | SPO2_F  SPO2_R | GCGCTTATGCTGTGCTACAA  GTACGGCTGGAAGGAAGTTG | This study |
| *SPO11-3* | Type II DNA topoisomerase VI subunit A (SPO11) homolog | KY224382 | SPO3_F  SPO3_R | GAGGACCGCTTCTACAACGA  GCCCTTCATGTAGACGGAGA | This study |
| *DMC1* | Meiosis-specific homolog of Rad51; promotes interhomolog recombination | KY224383 | DMC1_F  DMC1_R | CAGCAGAAGCTCTCCCAGAT  GTAGAGGCGAAGGTTGACCA | This study |
| *HOP1* | Protein that binds DSBs and oligomerizes early during meiotic prophase, and forms axial and lateral elements of the synaptonemal complex | KY224384  KY224385 | HOP1_F  HOP1_R | GTGGCATGAGGCTGCTCTAC  ACCTTGCCAACCATGAAGAA | This study |
| *MER3* | Meiosis-specific DEAD-box helicase that promotes Holliday junction resolution with crossover interference | KY224386  KY224387 | MER3_F  MER3_R | GACTCGGGCATGATTGAGAT  GAAGAGGGTCATCGTCTCCA | This study |
| *MND1* | Functions after meiotic DSB formation, and required for stable heteroduplex DNA formation | KY224388 | MND1_F  MND1_R | CTCGAGGCCGACAAGCTC  GATGAACTTCTTGCGCACCT | This study |
| *MSH5* | Forms a heterodimer with Msh4, interacts with Mlh1/Mlh3 heterodimer. Directs Holliday junction resolution toward crossover with interference | KY224389 | MSH5_F  MSH5_R | GACGGCATCTCCTTCCTGTA  TTCTTCTCGAGCATGACCTG | This study |
| *FLAG4* | Flagella dynein light chain TCTEX1; axonemal dynein | GS08244 | FLAG4_F  FLAG4_R | ACGTGTGCGATAATGCAGAA  GTCAGATCGAGAGGCCAAAG | [23] |
| *FLAG5* | Flagellar outer arm ODA-DC3; microtubule-associated with the dynein arm of flagellar axoneme | GS04411 | FLAG5_F  FLAG5_R | GACAGTACGGCCACGAGTTT  GCCGCTAGCTGCTGTAGTTT | [3] |
| *FLAG8* | Flagellar dynein heavy chain 2 (DHC2) – axonemal dynein | GS00012 | FLAG8_F  FLAG8_R | TACATCGACGGCTTCTTCCT  GGCACTCGTAGTCCTTCTCG | This study |
| *FLAG11* | flagellar inner arm intermediate chain | GS00514 | FLAG11_F  FLAG11_R | TACGACGCTCCATATCCACA  TCTGGTCCTTCTCGCTGTTT | This study |
| *PHOTO1* | Photoreceptor protein variant | GS00132 | PHOTO1_F  PHOTO1_R | ACGAGATTCGAGAGGCGATA  GCCACAAAGTAGCGACACAA | [3] |
| *PHOTO2* | Photoreceptor protein variant | GS00920 | PHOTO1_F  PHOTO1_R | CTTCGAAGTCTCCAGCCAAG  TCCTCCGAGTACACGCTCTT | [3] |
| *MYB* | MYB transcription factor | GS00273 | MYB_F  MYB_R | AGTGAGTTTCCTGCCTGCAC  CTATCTCCGCCACACTCACA | [3] |
| *H2A* | Histone protein | GS10455 | H2A_F  H2A_R | CGAGGAGCTCAACAAGTTCC  GAAACACCTGTGGGGAGAGA | [3] |
| *Actin* | Cytoskeleton protein  (used as housekeeping gene for data normalization) | *S64188.1  *S64193.1  *S64192.1  *S64191.1  *S64190.1  *S64189.1 | Actin_F  Actin_R | GACCGACTGGATGGTCAAG  GCCAGCTTCTCCTTGATGTC | [4] |
